# Idiopathic left-sided diaphragmatic hernia: a rare clinical image

**DOI:** 10.11604/pamj.2022.42.67.35121

**Published:** 2022-05-24

**Authors:** Anam Rajendra Sasun, Rashmi Ramesh Walke

**Affiliations:** 1 Department of Cardio-Respiratory Physiotherapy, Ravi Nair Physiotherapy College, Datta Meghe Institute of Medical Sciences (DU), Sawangi Meghe, Wardha, Maharashtra, India

**Keywords:** Diaphragmatic hernia, idiopathic cause, computed tomography

## Image in medicine

Idiopathic diaphragmatic hernia, which occurs without a traumatic etiology, is a very unusual condition with a wide spectrum of medical symptoms. Cough, chest pain, dyspnea, upper abdomen pain, bowel bladder problems, and vomitus are some of the common respiratory and gastrointestinal symptoms. To minimize life-threatening morbidity and fatality, surgery is required. This is a case of idiopathic left-sided diaphragmatic hernia which is an extremely rare condition. A 60-year-old male with no history of trauma reported to have been experiencing pain in the left upper abdomen along with breathing difficulties for the past 15 days. The pain was insidious in onset and progressive. On physical examination, chest expansion revealed differences of 2 cm and 1 cm each along with tenderness in the epigastric area. On auscultation, air entry was reduced bilaterally over lung fields. On investigation computed tomography (CT) impression of the abdomen and pelvis showed: 1) Large defect noted in left crus of the diaphragm with herniation of stomach to the left thoracic cavity; 2) There is a shift of mediastinum towards the right side; 3) Herniation of abdominal contents into the thoracic cavity; 4) Mild left-sided-pleural effusion with the consolidation of the left basal lung.

**Figure 1:**
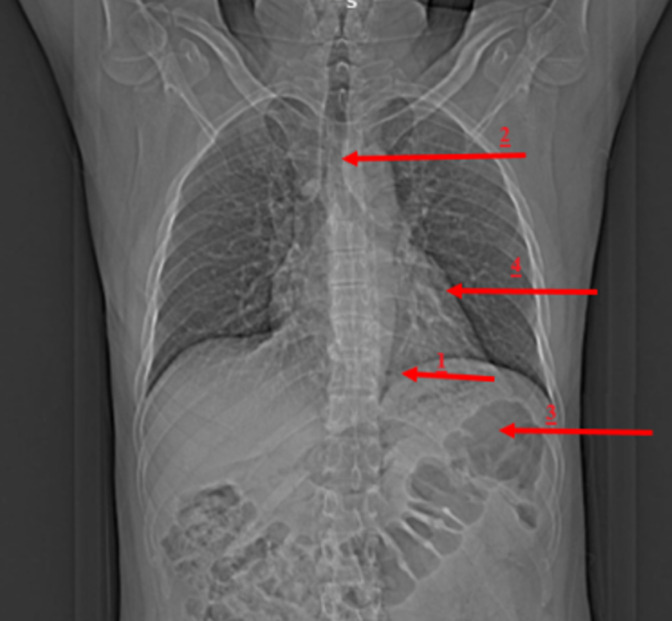
CT image of depicting herniation of abdominal contents into the thoracic cavity and shift of mediastinum towards right side

